# The Functions of a Medical Milk Commission

**Published:** 1912-11

**Authors:** Clarence Rhodes

**Affiliations:** Atlanta, Ga.


### THE FUNCTIONS OF A MEDICAL MILK COMMISSION.

By Clarence Rhodes, M. D., Atlanta, Ga.

In presenting this subject the writer wishes to call attention to a few of the many important functions of a Medical Milk Commission.

First, let us speak of the educational function, as it is through this feature of the commission that much good can be accomplished; in that it arouses the profession and laity to their full share of responsibility in this great movement.

It is our duty as physicians when we know a certain food is the cause of disease and the spreading of diseases to arouse from our lethargy and bestir ourselves and begin at home so to speak, in the Fulton County Medical Society to arouse interest among our own ranks in the Pure Milk Movement. A movement which is calling forth the best thoughts and efforts of the leading men in pediatric medicine in the world to-day, and the man or men who do not heed the call and keep abreast of the times in this movement is a drone or "back number" if he expects to practice pediatrics and hold the respect of his fellow practitioners.

Now, may I ask what has our society done in the last few years to aid in securing a pure milk supply for our city and patients?

Gentlemen, the truth hurts, but nevertheless it should be spoken, and it is simply this: that the men in our society who should be most interested in the pure milk movement and be up in arms fighting for "Certified Milk" for our babies and patients, are on the other side of the fence lending their energies in the defense of the putrid supply that we now have with our high infant mortality statistics staring them in the face. Is there a dairy furnishing milk to the city of Atlanta that you could truthfully say is perfectly safe for infant feeding? One whose cows have been tested and are free from tuberculosis or are regularly inspected by a competent veterinarian, or whose employees are inspected by a compenitent physician and are kown to be free from contagious and infectious diseases, and the milk produced to contain less than 10,000 bacteria per cc? I venture to say there is not one.

It is the prevailing opinion among the ranks of those most interested in this work, that at the present time it is impossible to get a safe and pure milk fit for clinical purposes in any city without strict medical supervision.

Has it ever been brought to your attention that a contaminated milk is consumed before it can be reported by the health authorities? This, it is true, might occur even with certified milk, but where there is a strict medical supervision this risk is reduced to a minimum.

It is a well established fact, that in every city where there is certified milk, the physicians and the laity have been aroused to its importance, and have carried on crusades and educational movements in the cause of pure milk; also the health departments have bestirred themselves in bettering the general milk supply for their various cities, and that the pure milk movement has been the prime factor in reducing infant mortality.

We hear so much now-a-days about pure food and drug laws. Is there a pure food law regulating the production of milk in our state? What food is more used and is of more vital importance to so many human lives? Still no effort is being made to remedy this condition.

Is it not time for us to take steps towards obtaining a pure milk for clinical purposes? Let us arouse ourselves and the laity to the importance of this movement, and call to our assistance the daily papers, the various women's clubs and organizations; the Chamber of Commerce would aid us and our public spirited citizens would come to our assistance. Others have done it; others are doing it, and why not Atlanta? May I venture to mention the cause? The members of the Fulton County Medical Society are not informed on this subject. Now let us inform ourselves and start this pure milk movement, and find out what is what and why in regards to milk fit for clinical purposes and get it.

Not only is the function of a medical milk commission educaticnal, but also economic, and it is in this field that our work is most productive. The reduction of infant mortality has been an economic problem that has puzzled statesmen and crowned heads of the European countries for generations and it behooves us to take warning and fortify ourselves against the ravages of infant mortality.

It has been demonstrated in cities where the pure milk movement is well under way that infant mortality has been greatly reduced; a few statistics will suffice to show this. In this country, in cities where the general infant mortality was from 20 to 25 per cent., was reduced as follows under the pure milk movement in 1909: Babie's Milk Fund Association, Baltimore 8 per cent.; Dispensary Hospital, Cleveland, 7.3 per cent.; Babie's Milk Fund Association, Louisville, 6 per cent.; New York Diet Kitchen, 2.75 per cent.; Milk and Baby Hygiene Association, Boston, 2.5 per cent.

Have we ever sat down and estimated the economic side of this question? I fear not or we would be striving for better conditions. Think of the financial loss to a nation in the death of one child. During the child bearing period the productive powers of the mother are decreassed. She suffers the tortues of the labor pains for what purpose? What has been gained by the individual or the nation if the infant is lost? Nothing but the financial loss to both has been heavy.

Gentlemen, when we pay more or as much attention to the rearing of children as we do to the raising of dogs, hogs, horses, cattle and sheep it will be a great financial gain to our country; for a nation's wealth is estimated by the numbers, health and intelligence of its people. Let me illustrate my point by referring to a few of the appropriations made by our legislature for 1911-'12.

For protection of live stock from contagious and infectious diseases and tick eradication $5,00.00 which was increased by the legislature this year to $15,000.00.

For the manufacturing and distributing hog cholera serum $5,000.00, which was increased to $6,000.00 annually by the last legislature. For State Board of Health $27,000.00, now note no increase in this appropriation was made by our last legislature, and think of the work this department is doing for the betterment of mankind in the manufacturing and free distribution of diphtheria antitoxin, in the Pasteur department, in the eradication of hook worm disease, typhoid fever and other phases of health work. The hogs and cattle of Georgia received more consideration accordingly than the people. Ought this to be the case? You will agree with me that this was money well spent from an economic standpoint. But I could find no record where any appropriation was made for the inspection or eradication of tuberculosis in our dairy stock, wherein lies a menace to the lives of all artificially fed children.

Is it not time for us to realize our duty as physicians and awaken to the responsibility that rests upon our shoulders, and demand that reforms be made in this direction? We have the power to accomplish this and thereby reduce the infant mortality in our state and city; all we would have to do is to realize our importance in the community and act accordingly.

No one who thinks, can deny the value of clean milk, milk produced from healthy cows, by clean methods, and showing a low bacterial count as an economic factor in that it reduces infant mortality more than any other factor at our command.

A great howl will be raised at the 5 or 6 cents increase in the price of certified milk, but when we take the trouble to point out that the use of such milk will prevent sickness and doctor bills, and that one of our visits will cost more than the added cost of certified milk for a month; then, and then only will the laity awaken to its importance.

We never stop to count the cost, beef juice so extensively used by the pediatrician and general practioner is an expensive food, it takes two quarts of beef juice to equal 5 oz. of certified milk in caloric value. We will pay from 30 to 40 cents a dozen for fresh eggs, when it takes more than six eggs to equal the food value of one quart of certified milk which will cost from 14 to 16 cents. We demand the best meats, fruits and vegetables, while "any old kind" of milk will do; milk is milk to the average person no matter if it is chalk and water.

The price of no article of food has remained so low as milk, which shows only an increase of one cent a quart in 20 years, when the price of cows, feed, labor and delivery methods have increased enormously. It is impossible for the dairyman to furnish a better product at the price paid. Let us be willing to pay a good price for a good product and then, only will certified milk assert its economic value upon the community. It is always better to study the cause than to treat the effect.

Other functions of a medical milk commission which could be dwelt upon at some length if time permitted, which will have to be passed over hurriedly. The philanthropic side of this question deserves mention, in that it has appealed to the wealthy in such a manner that they have responded nobly to the call of suffering humanity, and out of their abundance have founded milk dispensaries and milk depots in various cities for the uplift of the poor and needy.

The humanitarian of altrustic feature comes in for their full share of consideration with much food for thought, and no one can deny that it is through these avenues that we can appeal to the most people.

Last but not least, let me call your attention to the scientific side of this question, and it is in this feature that we physicians should most delight, for here we have the opportunity to put into practice our chemistry, biology, bacteriology, asepsis, preventive medicine and general hygiene. Should we not feel proud of the opportunity that this subject affords us, and get busy and acquit ourselves like men, and show to the South that we physicians of the good old State of Georgia, the "Empire State of the South" have seen the vision of the possibilities in preventive medicine which lie before our very threshold, and try at least to institute some reforms for the betterment of our milk supplies and dairy herds.

---

*Dr. F. G. Hodgson*—This paper or Dr. Rhodes has called our attention to a most important subject—that is—the prevention of infant mortality. A number of the larger cities are doing splendid work along this line with their societies for the prevention of infant mortality, their mothers clinics, their free milk depots, free ice depots, etc. Atlanta is behind in this sort of work and our society should be more actively interested in this sort of work.

I consider that parts of this paper are a little strong in their criticism. The milk supply of Atlanta hardly deserves to be called "putrid." Eight years ago this might have been true, but there has been a great improvement in our milk supply due to the efforts of Dr. Claude A Smith. It is a great deal purer and cleaner than it formerly was. We should not, however, sit down and pat ourselves on the back and be satisfied. There is room for improvement and we should continue to strive for still better milk. If a medical milk commission will insure us better and purer milk, then by all means let us strive to have one.

*Dr. Roy Blosser*—Our City Bacteriologist has done good work in improving the general milk supply for Atlanta and I am sure that Dr. Rhodes does not in the least discredit the good that has been done in this way. But it is not to be expected high standard desired for infants feeling. Therefore the need that the entire milk supply of a city can be brought up to that of special dairies under the supervision of a board of physicians to supply a milk of high quality for infant feeding. There is no reason why our general milk supply should suffer because we also have a milk of higher purity or certified milk, as it is called The work of the milk commission is not intended to supplant that of the City Bacteriologist and milk inspectors.

Many other cities have tried this plan and found that it resulted in a reduction of infant mortality.

*Dr. W. W. Adkins*:—I have enjoyed very much Dr. Rhodes' paper and the discussion. I have not been in Atlanta long enough to be in a position to intelligently discuss the merits of its milk supply, but feel sure that in not having certified milk, it has not milk that is good enough with which to feed babies. Dr. Rhodes' paper was probebly a little strong on some points, but taken as a whole I am of the opinion that he has hit the nail on the head. I had the pleasure of working with Dr. Rhodes in a New York hospital, where we ran a census during the winter months of seven and eight hundred, seventy-five of which were children. I am thoroughly convinced of his ability in infant feeding and that he is well posted on the subjects in his paper. As to the additional price of certified milk, I do not think very many people will complain of that, when they understand that it is for a better grade of milk, and the only proper kind with which their babies should be fed. During my service in the childrens' clinic at the Cornell dispensary I never saw a failure with a feeding case in which the instructions of Dr. Winters were carried out. He specifically instructed every mother to use only Bordens certified milk, and the dispensary sent out nurses to follow up all feeding cases to see tha all his instructions were carried out to the letter. These were all charity cases, and I never heard any of them complain at the additional price they had to pay to get certified milk.

Atlanta is looked upon and recognized all over the country as the leading city and the metropolis of the South; Atlanta boasts of its progressiveness, and it is progressive, but is far behind the times in not having certified milk. There is a great deal of charity done in this city, and I feel sure if the citizens were called upon would willingly respond with whatever financial aid was necessary to get certified milk; but someone has to start the movement, and that someone should be the leading medical organization, the Fulton County Medical Society.

All public dairymen should be carefully watched or they are liable to grow careless with their stock and their methods of handling the milk, they are not in business for their health, and the Atlanta dairymen are not any different from those of other cities. We undoubtedly have a most efficient and competent city bacteriologist, but he cannot do it all, though he is to be complimented on his earnest efforts to better the milk supply, and deserves much credit for what he has already accomplished according to the discussions. The people of Atlanta want the best, and seem willing to pay for it; certified milk is the best to be had and they should have it, and it is most assuredly up to the Fulton County Medical Society to see that they get it.

*Dr. L. B. Clarke*:—The position I am going to take is one easily misunderstood, so, I will preface it by saying, that I am not opposed to medical milk commissions; for, no man recognizes more than I do, the great benefits that have been accrued to humanity and the markedly lessened infant morality brought about through this splendid work in many parts of the country, where impure milk was previously sold. Furthermore, the great necessity for continuing the work begun by Dr. Coit, in many sections and cities where exists a contaminated milk supply, but, I do contend that a milk commission is not necessary in Atlanta now, and, that the sweeping attack of Dr. Rhodes upon the milk supply of this city is not alone entirely unwarranted, but does a great injustice to the city, to those whose official duty it is to overlook our milk, and to the physicians who are using it, and I protest against it.

Time was, when a condition of affairs practically existed here, when, as in other cities of the North and East, almost anything was sold for milk, but that is past history. Perhaps there are gentlemen present who will remember the fight against our milk conditions of several years ago, an active interest in which was taken by this society. It will be remembered that I had the honor to read before this body a paper upon this subject during that fight, and that the society in the interests of public education, instructed that the paper be published in the daily press. In that paper, I called attention among other matters to a pasteurizing plant established here which disbursed milk and all over the city, and created a sense of false security for the milk was either sour when delivered, or, as many families complained would *never sour,* the latter due, of course, to preservatives it contained.

At the time referred to above, infant mortality in Atlanta was very high, and I demonstrated this in a report to this cosiety tabulated from the records in the offices of the Board of Health. Particularly was this true during the summer months, the time of the Board of Health. Particularly was this true during the summer months, the time of prevalence of intestinal disease.

This has all been changed, thanks to the close watchfulness and earest efforts of our capable city bacteriologist, Dr. Smith, and to the strict law we have enacted, governing the sale of milk.

From a practical, daily experience with artificially fed babies, I pronounce tthe milk of Atlantta good, and perfectly fit for food. It New York City it is still sterilized. I neither pasteurize nor sterilize for my formulae, but, feed it raw, and I do not have the fatalities Dr. Rhodes charges to our milk. The fact is, I have especially noted during the present summer and the summer of 1911, the almost total absence of Ileo-colitis and Gastroenteric infection in my work among raw milk babies; and now, that I think of it, I cannot recall a single case of either disease occurring during the past summer in my own work, and have only seen cases brought from out of the city, or in consultation on the outskirts, where other agencies than milk had to be included as causative factors.

Again, the cases of intestinal disease that I have seen during the past few years in my work or in consultaitno, have generally been mild, and bear no relation to the intense infections common in this city five to seven years back. The most closely associated disease with impure milk, is cholera-infantum. I have never seen but one case here, and that was years ago. If our milk supply were as claimed, putrid and unfit for food, this disease would be as common as colds and coughs. As it is, local preventive measures have placed it among the rare diseases.

These statements show that our milk supply is not alone not putrid, but, that rather, it should be lauded.

Dr. Rhodes presents no statistics in support of his statements and has evidently not examined the Health Board of Records, which will not bear out the mortality claims he presents.

It must beremembered that our milk delivery conditions differ widely fro mthose of the large eastern cities. Medical Milk Commissions demand that milq shall be delivered to the consumer before it is twenty-four hours old. It Atlanta, the milk is produced with but few exceptions, from dairies locatted six to twelve miles from the city, delivered to the consumer a few hours after milking, and is almost *consumed* by the child *before* it is twenty-four hours old.

An important fact connected with the souring of milk is the lack of public education in caring properly for milk after it has been delivered to the home. Many sins charged to the dairymen lie at the door of the consumer. Time and again have I seen bottles of milk on front porches, exposed to the rays of hot summer sun, probably been there for hours, and bacteria multiplying. Many people, even in hot weather, do not place milk in the refrigerator promptly, and many refrigerators are valueless in rettaining milk at the temperature at which our *city laws force the dairymen to deliver it.*

The poor man is justified in desiring milk as pure for his baby as can be obtained by the rich man. As it is, he can readily obtain it for the same restrictive laws apply to all dairies doing business in Atlanta. The only ouscome that I can see from a milk commission here, is to raise the price and make it prohibitive among the very class of people who most need it, and among whom disease is not common.

I have recently taken charge of an infant from New York City, who under an eminent peditrist was fed sterilized milk there, and who is thriving on Atlanta milk, fed raw.

I believe in milk commissions where necessary, but they are intended to meet conditions other than those we have in Atlanta. Let us keep up the standard, and have pure milk for all at the same price.

*Dr. C. A. Rhodes*:—In closing this discussion, I wish to assure Dr. Smith that I did not mean to cast any reflection upon his noble work in taking care of Atlanta's milk supply, for he deserves to be congratulated for the good work he has done. But my remarks were more especially directed to those of the Fulton County Medical Society who should be most concerned, particularly the pediatrists who are making no effort to better our milk condition, but are sitting still, contented to let Dr. Smith do all the work.

Dr Clark says he is not opposed to a Medical Milk Commission. What I want to know is he in favor of one? There is no half-way position to be taken on this subject.

I was surprised some time ago, when Dr. Smith read a paper on "The Care of Milk For Infant Feeding" to hear a professos of pediatrics ask Dr. Smith if he had tested any of these "Baby Refrigerators." Gentlemen, this shows the attitude of those who should be most interested in the "Pure Milk Movement." They are willing to sit still and let others do their work and they even ask the City Bacteriologist to do the work that they as individuals should be progressive enough to do themselves.

Dr Clark's argument against "Certified Milk" on account of the increased price of the product, is indeed a lame one. Because Mr. Jones is wealthy and his neighbor Mr. Smith is very poor, is that any argument why Mr. Jones' baby should not have certified milk just because Mr. Smith is not able to buy it for his?

Dr. Clark says the milk of Atlanta is fit for infant feeding. Gentlemen, I feel sorry for the infants who will be fed by those who think that Atlanta's milk is fit for infant feeding.

In closing this discussion I wish to say out of all the smoke that has been created this evening I see the glimpse of a spark which will kindle into a flame, and within five years Atlanta will have certified milk whether our honored president is opposed to it or not.

